# From dysbiosis to malignancy: decoding gut-driven pathways to clinical management in hepatocellular carcinoma

**DOI:** 10.3389/fcimb.2026.1852380

**Published:** 2026-06-09

**Authors:** Abdulrahman Ismaiel, Mhd Bashir Almonajjed, Mahdi Wardeh, Ahmed Abdelghafar, Stefan-Lucian Popa, Cristina Sabo, Dan L. Dumitrascu

**Affiliations:** 1 2nd Department of Internal Medicine, “Iuliu Hatieganu” University of Medicine and Pharmacy, Cluj-Napoca, Romania; 2 Faculty of Medicine, “Iuliu Hatieganu” University of Medicine and Pharmacy, Cluj-Napoca, Romania

**Keywords:** biomarkers, dysbiosis, gut-liver axis, hepatocellular carcinoma (HCC), immunotherapy, leaky gut, metabolites, microbiome

## Abstract

Hepatocellular carcinoma (HCC) is undergoing a profound global epidemiological shift, transitioning from viral-driven etiologies to metabolic dysfunction-associated steatotic liver disease (MASLD). This transition challenges traditional cirrhosis-centric surveillance, as a significant proportion of MASLD-HCC develops in non-cirrhotic livers. Parallel to these metabolic shifts, the gut-liver axis has emerged as a central orchestrator of hepatocarcinogenesis. This review decodes the complex gut-driven pathways fueling HCC, highlighting the oncogenic consequences of structural and functional dysbiosis. Dietary patterns and etiology-specific microbial shifts compromise the intestinal and gut-vascular barriers, precipitating a structural “leaky gut”. This disruption facilitates the robust translocation of pathogen-associated molecular patterns (PAMPs), particularly lipopolysaccharide (LPS), and toxic microbial metabolites like secondary bile acids, specifically deoxycholic acid, into the portal circulation. Consequently, hepatic innate immunity is chronically activated via Toll-like receptor 4 (TLR4) signaling on Kupffer and hepatic stellate cells, fostering metainflammation, cellular senescence, genomic instability, and a highly immunosuppressive, pro-tumorigenic microenvironment. Furthermore, the depletion of keystone commensals diminishes the protective reservoir of short-chain fatty acids (SCFAs), exacerbating oncogene activation. Translating these mechanistic insights into the clinic, we explore the utility of distinct microbial signatures and metabolomic profiles as non-invasive diagnostic biomarkers. Such tools are urgently needed to bridge the early-detection gap in the expanding MASLD demographic. Finally, we discuss the pivotal role of the microbiome in modulating responses to immune checkpoint inhibitors (ICIs), notably through immune-stimulating taxa like *Akkermansia muciniphila*, and outline emerging gut-targeted therapies, including next-generation probiotics and fecal microbiota transplantation, aimed at restoring host-microbiome homeostasis to prevent and manage HCC. By decoding these gut-driven pathways, this review provides a comprehensive framework for integrating the microbiome-onco axis into precision oncology, offering novel avenues to combat the rising global burden of hepatocellular carcinoma.

## 1 Introduction

Hepatocellular carcinoma (HCC) remains one of the most formidable challenges in global oncology, characterized by a complex interplay of genetic, environmental, and microbial factors. Currently ranking as the third leading cause of cancer-related mortality worldwide, the disease exacts a profound socioeconomic toll and necessitates continuous refinement of our diagnostic and therapeutic frameworks (Philips et al., 2021; Fu et al., 2025; Monti et al., 2025). Historically, the clinical management of HCC has been deeply intertwined with the surveillance of established cirrhotic populations (Philips et al., 2021; Fu et al., 2025; Monti et al., 2025). However, the global epidemiological landscape of this malignancy is currently undergoing a rapid and profound transformation (Philips et al., 2021; Fu et al., 2025; Monti et al., 2025).

Although hepatitis B virus (HBV) and hepatitis C virus (HCV) have long been the primary global drivers of HCC, their relative contribution is steadily decreasing, largely due to successful vaccination campaigns and the availability of potent direct-acting antivirals (Banerjee et al., 2024; Monti et al., 2025; Ren et al., 2025). Conversely, metabolic dysfunction-associated steatotic liver disease (MASLD), fueled by the global epidemics of obesity and metabolic syndrome, has emerged as the fastest-growing etiological driver of liver cancer (Chen Z. et al., 2025; Jin et al., 2025; Monti et al., 2025). Projections suggest that the incidence of metabolic dysfunction-associated steatohepatitis (MASH)-related HCC may surge by 122% in the United States between 2016 and 2030 (Philips et al., 2021). This etiological shift severely complicates clinical surveillance; roughly 25% to 50% of MASLD-associated HCC cases develop in non-cirrhotic livers, effectively bypassing traditional screening programs (Philips et al., 2021; Jin et al., 2025; Monti et al., 2025). Furthermore, hepatic steatosis and subcutaneous adiposity heavily attenuate sound waves, rendering standard ultrasound screening significantly less effective in this growing patient demographic (Jin et al., 2025; Monti et al., 2025).

Parallel to these metabolic shifts, the gut-liver axis has been identified as a critical determinant in hepatocarcinogenesis. The liver receives approximately 70-75% of its blood supply directly from the intestine via the portal vein, exposing hepatic tissue to high concentrations of gut-derived signals (Liu and Yang, 2023; Monti et al., 2025; Xiao et al., 2025). In the context of dietary triggers like high-fat or high-fructose diets, structural dysbiosis compromises the intestinal and gut-vascular barriers (Daniel et al., 2024; Monti et al., 2025; Xiao et al., 2025). This “leaky gut” permits the unchecked translocation of pathogen-associated molecular patterns (PAMPs), such as lipopolysaccharide (LPS), and toxic metabolites, including secondary bile acids like deoxycholic acid (DCA) (Chen Z. et al., 2025; Monti et al., 2025; Wang et al., 2025c). These factors overwhelm the liver’s natural endotoxin tolerance, activating innate immunity via Toll-like receptor (TLR) signaling on Kupffer cells and hepatic stellate cells (Komiyama et al., 2021; Liu and Yang, 2023; Daniel et al., 2024). This cascade provokes a relentless cycle of metainflammation, cellular senescence, and genomic instability, ultimately generating a highly permissive, pro-tumorigenic niche (Xu et al., 2021; Li et al., 2025; Monti et al., 2025).

Recognizing the central role of the microbiome in tumor progression has opened novel avenues for clinical intervention, particularly through specific microbiome modulation. The identification of distinct microbial signatures and non-invasive biomarkers offers immense potential to circumvent the limitations of traditional imaging in diagnosing early HCC across various geographic locations (Yang et al., 2023; Li and Liu, 2025; Li et al., 2025). Beyond diagnostics, modulating the gut microbiome is a critical determinant of therapeutic efficacy, particularly regarding the response to immune checkpoint inhibitors (ICIs) (Ponziani et al., 2022; Chen PJ. et al., 2025). Interventions such as next-generation probiotics and fecal microbiota transplantation (FMT) are actively being explored to restore homeostatic balance, enhance anti-tumor immunity, and prevent disease progression (Zhu et al., 2024; Xiao et al., 2025).

Given this rapid etiological evolution, there is an urgent need to synthesize how metabolic shifts and microbial dysbiosis cooperatively influence tumor progression and clinical outcomes. Although a number of recent reviews have established broad associations between the gut microbiome and liver cancer, many of them concentrate on traditional viral etiologies or purely cirrhosis-driven models. This review is unique in that it is the first to explicitly discuss the rapid epidemiological shift towards MASLD-associated HCC, with a specific focus on hepatocarcinogenesis in non-cirrhotic livers, a rapidly growing population that currently bypasses standard surveillance protocols. We also make a unique contribution in linking complex molecular mechanisms like dysbiosis-induced leaky gut and metabolic signaling to immediate clinical translation. This review critically appraises the translational readiness of non-invasive microbial biomarkers and next-generation gut-targeted therapies to deliver a highly tailored, practice-oriented framework for the management of HCC in the post-viral era. Therefore, this narrative review aims to comprehensively explore the mechanisms by which the gut-liver axis mediates HCC. Furthermore, we evaluate the significant challenges of current surveillance strategies in the post-viral era and discuss the transformative potential of microbiome-based biomarkers and targeted therapeutic modulations to bridge the gap in early detection and precision management across diverse global populations.

## 2 Pathophysiology

### 2.1 Pathogenesis: etiology-specific nuances

Chronic viral hepatitis causes dysbiosis by affecting liver processes such as bile salt secretion and albumin production (Liu and Yang, 2023). Because BAs generally have antibacterial properties, decreased production causes bacterial overgrowth and structural alterations in the microbial population (Liu and Yang, 2023). This impaired liver function also induces intestinal wall edema, which weakens the mucosal barrier and results in a “leaky gut” (Liu and Yang, 2023). Increased permeability permits bacteria and their metabolites to enter the portal vein and proceed to the liver (Komiyama et al., 2021; Wang J. et al., 2025). Specific bacteria, such as *Enterococcus*, are considerably elevated in individuals with HBV-related HCC and cirrhosis. This pro-inflammatory genus causes intestinal leakage and has been found in HCC tumor tissues (Feng et al., 2025). Cytolysin-positive *Enterococcus faecalis* moves from the gut to the liver, a process that has been linked to increased patient mortality. Furthermore, *Bacteroides* species are overrepresented in HCC patients versus those with cirrhosis alone (Song Q. et al., 2023; Scarpellini et al., 2024). *Bacteroides* abundance is associated with higher levels of pro-inflammatory cytokines such as IL-8 and IL-13 (Scarpellini et al., 2024). Furthermore, *Bacteroides* is related with an increase in MDSCs, which promotes tumor development by creating an immunosuppressive environment (Scarpellini et al., 2024). These translocated bacterial components, such as LPS, interact with TLR4 and TLR9 on hepatic Kupffer cells (Liu and Yang, 2023; Kim et al., 2025). This activation activates the NF-κB pathway, resulting in the large production of TNF-α and IL-6 (Liu and Yang, 2023; Kim et al., 2025).

Alcohol and its metabolite, acetaldehyde, raise intracellular calcium ions in gut epithelial cells, compromising tight junction integrity (Wang J. et al., 2025). This disturbance enhances gut wall permeability, allowing dysbiotic bacteria and endotoxins such as LPS to enter the portal vein in large quantities (Wang J. et al., 2025). While alcohol uniquely destroys these tight junction complexes, acetaldehyde’s mechanism is based on calcium regulation rather than direct protein breakdown (Kim et al., 2025; Wang J. et al., 2025). Chronic alcohol intake also depletes the populations of *Lactobacillus* and *Bifidobacterium* (Wang J. et al., 2025). This depletion is due in part to alcohol-induced oxidative stress and ethanol’s direct adverse impacts on the gut environment (Kim et al., 2025). In comparison with viral hepatitis, when *Bifidobacterium* may be abundant, its absence is a distinct feature of alcohol-related liver disease (Scarpellini et al., 2024; Marawan et al., 2025). In preclinical murine models, depletion of *Bifidobacterium pseudolongum* is directly connected to HCC development because it lowers acetate levels in the liver (Song Q. et al., 2023; Wang J. et al., 2025). These *in vivo* studies demonstrate that without acetate to bind GPR43, the oncogenic IL-6/JAK1/STAT3 signaling cascade goes uncontrolled (Song Q. et al., 2023; Wang J. et al., 2025). Similarly, preclinical *in vitro* and *in vivo* studies suggest that the loss of *Lactobacillus acidophilus* eliminates valeric acid, which typically inhibits the Rho-GTPase pathway and causes cell cycle arrest (Kim et al., 2025). Reintroducing these bacteria, or utilizing probiotics such as VSL#3, has been found to diminish the growth and size of liver tumors (Scarpellini et al., 2024; Monti et al., 2025).

Even before considerable liver damage develops, a high-fat diet affects the GVB (Monti et al., 2025). This disturbance is characterized by an increase in PV1, a measure of endothelial cell permeability (Monti et al., 2025). In addition to disrupting GVB, these diets weaken the epithelial barrier by lowering the expression of tight junction proteins such as ZO-1 and E-cadherin (Zhang et al., 2021; Monti et al., 2025). Studies suggest that enhancing barrier function with metabolites such as acetate occurs before tumor suppression, supporting the barrier deficiency as an early cause of the illness (Song Q. et al., 2023). The high-fat diet causes these failures by increasing the number of *Mucispirillum* and *Desulfovibrio* while decreasing *Bifidobacterium* (Zhang et al., 2021). This dysbiosis causes a decline in beneficial metabolites such as 3-indolepropionic acid (IPA) and short-chain fatty acids (SCFAs) (Zhang et al., 2021; Ren et al., 2025). Metabolic HCC is frequently associated with a high Firmicutes/Bacteroidetes (F/B) ratio (Monti et al., 2025). This ratio gradually increases as liver disease progresses from basic steatosis to NASH (Monti et al., 2025). A high F/B ratio is mostly caused by the loss of *Bacteroides*, which are essential for gut barrier integrity (Chen et al., 2023). Enriched Firmicutes, such as *Clostridium*, speed up the conversion of main BAs into secondary BAs like DCA in both human cohorts and animal models (Monti et al., 2025). In preclinical murine models, DCA functions as a carcinogen by causing DNA damage and cellular senescence in hepatic stellate cells (Monti et al., 2025). The F/B ratio has demonstrated potential as a biomarker; for example, it is considerably higher in HCC patients than in healthy controls (Hashem et al., 2025). This ratio is also associated with clinical decline, with favorable associations to direct bilirubin levels (Hashem et al., 2025). Interventions that restore a reduced F/B ratio, such as FMT, are linked to better liver function (Hashem et al., 2025).

### 2.2 Diet as a driver of regional shifts

Regional changes from viral-related HCC to MASLD-associated HCC are largely determined by dietary patterns (Philips et al., 2021; Fu et al., 2025; Monti et al., 2025). One of the main causes of gut dysbiosis and subsequent cancer is high consumption of cholesterol, fructose, and saturated fats, which are typical of Western dietary patterns (Daniel et al., 2024; Xiao et al., 2025). These diets cause dysbiosis, which is characterized by an increase in opportunistic pathogens like *Mucispirillum* and *Desulfovibrio* and a decrease in beneficial bacteria like *Bacteroides* and *Bifidobacterium* (Li et al., 2022; Xiao et al., 2025). The intestinal barrier is compromised by this dysbiosis, which increases LPS translocation and causes hepatic inflammation (Daniel et al., 2024; Xiao et al., 2025). In particular, high dietary fructose causes the gut microbiota to produce acetate, which stimulates hepatic lipogenesis and increases protein O-GlcNAcylation to support the growth of HCC cells (Yu et al., 2024a; Li et al., 2025). Furthermore, diets high in red meat cause microorganisms to produce trimethylamine (TMA), which the host then transforms into trimethylamine N-oxide (TMAO) (Banerjee et al., 2024). Regardless of cirrhosis status, elevated circulating TMAO is strongly linked to the risk of HCC and encourages hepatic fibrosis and inflammation (Banerjee et al., 2024; Li and Liu, 2025). On the other hand, diets high in fiber, whole grains, and vegetables encourage microbial diversity and the synthesis of anti-inflammatory SCFAs, though the function of SCFAs can vary depending on the situation (Rajapakse et al., 2023; Daniel et al., 2024; Li and Liu, 2025; Xiao et al., 2025).

### 2.3 Enterohepatic circulation and bidirectional regulation

There is a bidirectional functional relationship between the gut and liver (Chen Z. et al., 2025; Li et al., 2025; Wang J. et al., 2025). In order to preserve homeostasis, the liver secretes bile which contains primary BAs, immunoglobulin A (IgA), and antimicrobial molecules directly into the intestinal lumen. Bile transport is compromised in diseases such as cirrhosis, which lowers bile acid levels in the gut and promotes dysbiosis (Li et al., 2025). The enterohepatic circulation is the main mediator of the intestinal-liver interaction (Chen Z. et al., 2025). Gut bacteria convert primary BAs secreted by the liver into secondary BAs like lithocholic acid (LCA) and DCA (Chen Z. et al., 2025; Monti et al., 2025; Wang et al., 2025c). Through the portal system, these secondary BAs are reabsorbed and sent back to the liver (Acharya and Thirunavukkarasu, 2024; Li et al., 2025). Dysbiosis in HCC causes the accumulation of toxic, hydrophobic secondary BAs such as DCA, which cause DNA damage and generate a senescence-associated secretory phenotype (SASP) in hepatic stellate cells (HSCs) (Xu et al., 2021; Li et al., 2025; Monti et al., 2025). This trafficking also affects hepatic immunity; primary BAs increase the concentration of anti-tumor CXCR6+ natural killer T (NKT) cells on liver sinusoidal endothelial cells (LSECs) by upregulating CXCL16, whereas secondary BAs decrease CXCL16 to promote tumor development (Xu et al., 2021; Xiao et al., 2025).

### 2.4 Hepatic immune surveillance and the “first-pass” exposure

The portal vein provides around 70-75% of the liver’s blood supply straight from the intestine, exposing hepatic cells to large levels of gut-derived Pathogen-Associated Molecular Patterns (PAMPs) (Liu and Yang, 2023; Monti et al., 2025; Xiao et al., 2025). Under normal circumstances, the liver functions as a “firewall” in which Kupffer cells (KCs) and LSECs remove low amounts of microbial products without evoking a damaging immune response, a process known as “endotoxin tolerance” (Liu and Yang, 2023; Ohtani et al., 2023). Despite regular LPS exposure, healthy livers negatively control Toll-like receptor 4 (TLR4) signaling, preventing excessive inflammation. However, when the intestinal barrier is disrupted (“leaky gut”), the translocating load of PAMPs exceeds this filtering capacity, destroying hepatic tolerance and initiating long-term innate immune activation (Komiyama et al., 2021; Liu and Yang, 2023; Daniel et al., 2024). High LPS levels activate TLR4 on KCs, causing a huge release of pro-inflammatory cytokines such as IL-6, TNF-α, and IL-1, which promote hepatocyte proliferation while inhibiting apoptosis (Xu et al., 2021; Daniel et al., 2024; Xiao et al., 2025). Furthermore, LPS activates TLR4 on HSCs, causing the release of epiregulin, a hepatomitogen that promotes tumor survival (Wang et al., 2025c).

### 2.5 The role of keystone commensals and metabolite signaling

The onset of HCC is essentially connected to a decrease in microbial diversity and the loss of “keystone” commensals such as *Faecalibacterium prausnitzii* and *Bifidobacterium*, which decreases the liver’s anti-inflammatory tone (Yang et al., 2023; Zhang Y. et al., 2025; Guo et al., 2026). These commensals regulate hepatic homeostasis by generating SCFAs, such as acetate and butyrate (Xu et al., 2021; Song Q. et al., 2023; Jinato et al., 2024). *Bifidobacterium pseudolongum* generates acetate, which travels via the portal vein and binds to G protein-coupled receptor 43 (GPR43) on hepatocytes, effectively inhibiting the oncogenic IL-6/JAK1/STAT3 signaling cascade (Song Q. et al., 2023). As a result, the elimination of these microorganisms causes unregulated activation of IL-6 signaling (Song Q. et al., 2023). Similarly, *F. prausnitzii* is a significant producer of butyrate, which inhibits histone deacetylases (HDACs) and the NF-κB pathway in immune cells (Xu et al., 2021; Jinato et al., 2024; Goyal et al., 2025). Its depletion leads to increased production of pro-inflammatory cytokines such as TNF-α and IL-6 (Trivedi et al., 2023; Xiao et al., 2025). Furthermore, these keystones promote the expression of tight junction proteins such as ZO-1 and occludin; their absence leads to increased intestinal permeability (“leaky gut”), allowing LPS to enter the portal circulation and activate TLR4 on hepatic Kupffer cells (Rajapakse et al., 2023; Song Q. et al., 2023; Monti et al., 2025). Furthermore, the absence of *F. prausnitzii* inhibits regulatory T cell (Treg) differentiation, altering the immunological balance to a pro-inflammatory Th17-driven state (Xu et al., 2021; Jinato et al., 2024; Chen PJ. et al., 2025).

### 2.6 Pathobionts and the genotoxic environment

The overgrowth of pathobionts such as *Enterococcus* and Proteobacteria generates a genotoxic environment that promotes DNA damage (Acharya and Thirunavukkarasu, 2024; Marawan et al., 2025). In preclinical *in vitro* models, certain *Enterobacteriaceae* generate Cytolethal Distending Toxin (CDT), which causes double-strand DNA breaks and arrests cells in the G2/M phase (Acharya and Thirunavukkarasu, 2024). Furthermore, murine models demonstrate that E. coli strains that include the pks genomic island generate colibactin, which crosslinks DNA and leads to mutagenesis in the liver (Acharya and Thirunavukkarasu, 2024; Marawan et al., 2025). *Enterococcus faecalis* produces extracellular superoxide, which causes chromosomal instability (Liu and Yang, 2023; Acharya and Thirunavukkarasu, 2024). Furthermore, high-alcohol-producing *Klebsiella pneumoniae* strains create endogenous ethanol, which is converted to the strong carcinogen acetaldehyde, disrupting DNA repair (Ohtani et al., 2023; Goyal et al., 2025). Dysbiosis also enhances the conversion of main BAs into DCA, which causes mitochondrial malfunction and DNA damage in hepatic stellate cells, resulting in the SASP (Luo et al., 2022a; Capasso et al., 2025; Li and Liu, 2025) (**Table 1**. Demonstrates the specific mechanisms of pathogenic versus protective bacteria).

**Table 1:** Pathogenic versus protective bacteria in HCC.

Bacterium	Role	Specific mechanism	Molecular target	Impact on liver/tumor	Evidence tier/model	References
*Klebsiella pneumoniae*	Pathogenic	Surface protein interaction; Gelatinase secretion	PBP1B binds TLR4; Macrophage-derived MMP-2/-9	Activates oncogenic signaling; disrupts gut barrier to facilitate translocation	Preclinical (*In vivo* murine models) & Human observational	(Wang et al., 2025a)
*Catenibacterium mitsuokai*	Pathogenic	Secretion of oncometabolite; Surface protein adhesion	Quinolinic Acid (QA) binds TIE2; Gtr1/RagA binds -catenin	Activates PI3K/AKT pathway; disrupts gut barrier and colonizes liver cells.	Preclinical (*In vivo* murine models & *In vitro*) & Human observational	(Zhang Y. et al., 2025)
*Clostridium species*	Pathogenic	7-α dehydroxylation of bile acids	Secondary Bile Acids (DCA); suppression of CXCL16 on LSECs	Induces DNA damage/SASP in HSCs; reduces hepatic NKT cell accumulation (immunosuppression).	Preclinical (*In vivo* murine models) & Human observational	(Xu et al., 2021; Luo et al., 2022a; Song Y. et al., 2023)
*Enterococcus faecalis*	Pathogenic	Secretion of exotoxin	Cytolysin (GelE); TLR4	Causes direct hepatocyte death/lysis; increases gut permeability.	Preclinical (*In vivo* murine models & *In vitro*) & Human observational	(Li et al., 2025; Zhang Y. et al., 2025)
*Escherichia coli*	Pathogenic	Production of genotoxin	Colibactin; TLR4 (via LPS)	Induces double-strand DNA breaks (genomic instability); promotes inflammation.	Preclinical (*In vivo* murine models & *In vitro*) & Human observational	(Ohtani et al., 2023; Acharya and Thirunavukkarasu, 2024; Chen PJ. et al., 2025)
*Bifidobacterium pseudolongum*	Protective	Metabolite secretion	Acetate binds GPR43 (FFAR2)	Inhibits the IL-6/JAK1/STAT3 oncogenic signaling pathway.	Preclinical (*In vivo* murine models & *In vitro*)	(Song Q. et al., 2023)
*Lactobacillus acidophilus*	Protective	Metabolite secretion	Valeric Acid binds GPR41/43	Suppresses the Rho-GTPase pathway; induces cell cycle arrest.	Preclinical (*In vivo* murine models & *In vitro*)	(Chen Z. et al., 2025; Monti et al., 2025)
*Akkermansia muciniphila*	Protective	Membrane protein interaction; Mucin regulation	Amuc_1100 activates TLR2; Upregulates RegIII	Enhances gut barrier integrity; promotes NKT cell recruitment via IL-12.	Preclinical (*In vivo* murine models & *In vitro*) & Human clinical cohorts	(Capasso et al., 2025; Goyal et al., 2025; Monti et al., 2025)
*Lactobacillus rhamnosus* GG	Protective	Metabolite secretion	5-MIAA activates Nrf2	Protects against oxidative liver injury; downregulates bile acid synthesis.	Preclinical (*In vivo* murine models)	(Wang et al., 2025c)
*Odoribacteraceae*	Protective	Bile acid transformation	Isoallo-LCA	Induces Marco+ immunosuppressive macrophages to limit excessive inflammation.	Preclinical (*In vivo* murine models)	(Monti et al., 2025)

PBP1B, Penicillin binding protein 1B; TLR, Toll-like Receptor; MMP, Matrix Metallopeptidase; DCA, Deoxycholic Acid; LSECs, Liver Sinusoidal Endothelial Cells; NKT, Natural Killer T cells; SASP, Senescence-Associated Secretory Phenotype; HSCs, Hepatic Stellate Cells; GPR, G Protein-Coupled Receptor; 5-MIAA, 5-methoxyindoleacetic acid; Nrf2, Nuclear factor erythroid 2-related factor 2.

### 2.7 Molecular mechanisms of the “leaky gut” and gut-vascular barrier

The primary cause of increased permeability in HCC is the downregulation of TJ proteins such as Occludin, Claudin-1, and ZO-1 (Song Q. et al., 2023; Wang et al., 2025a; Zhang Y. et al., 2025). While tissue ZO-1 levels are low, higher serum levels of ZO-1 act as a biomarker for barrier breakdown (Rajapakse et al., 2023; Yang et al., 2023). The LPS-TLR4-MLCK signaling cascade is an important biological route: LPS binds to TLR4 on intestinal epithelial cells, triggering the MyD88-dependent pathway that controls Myosin Light Chain Kinase (Rajapakse et al., 2023; Li and Liu, 2025). This causes the perijunctional actomyosin ring to shrink, physically opening tight junctions (Rajapakse et al., 2023; Li and Liu, 2025). Furthermore, secondary BAs such as DCA operate as “pore-forming” detergents, physically disrupting the lipid bilayer (Luo et al., 2022a; Yoshikawa et al., 2023). Chronic consumption of alcohol physically expands paracellular gaps and damages the protective mucus barrier (Daniel et al., 2024; Chen Z. et al., 2025).

The GVB acts as a size-selective “second line” of defense, allowing only molecules up to 4 kDa (Chen Z. et al., 2025). When the GVB is damaged, Plasmalemma Vesicle-Associated Protein-1 (PV1) levels rise, indicating that interstitial spaces are expanding (Chen PJ. et al., 2025; Chen Z. et al., 2025). This break enables the direct transport of entire living bacteria into the portal circulation (Lin et al., 2025; Wang et al., 2025a). In preclinical murine models, *K. pneumoniae* stimulates macrophage-mediated gelatinase activity (MMP-2/9) to dissolve the extracellular matrix and transport live bacteria to the liver (Wang et al., 2025a). Similarly, animal studies reveal that E. coli C17 can directly access the GVB via virulence factor pathways, so contributing to a pre-metastatic niche (Chen Z. et al., 2025; Lin et al., 2025). This GVB breakdown allows for a significant flood of big PAMPs, such as LPS and peptidoglycans, that bypass the hepatic “firewall” (Luo et al., 2022a; Song Y. et al., 2023).

### 2.8 Bacterial translocation and endotoxemia

Translocated LPS activates the TLR4-NF-κB axis in Kupffer cells, resulting in the long-term release of pro-inflammatory cytokines such as TNF-α, IL-6, and IL-1β (Li et al., 2025; Xiao et al., 2025). These cytokines promote “metainflammation,” which is necessary for hepatocarcinogenesis (Rajapakse et al., 2023; Guo et al., 2026). LPS also causes HSCs to release epiregulin, a strong hepatomitogen that helps tumors survive (Rajapakse et al., 2023; Li et al., 2025). Chronic exposure to translocated LPS disrupts “endotoxin tolerance” in the liver, resulting in a chronic immunological response (Liu and Yang, 2023). Furthermore, LPS translocation activates Myeloid-Derived Suppressor Cells (MDSCs), which inhibit cytotoxic CD8+ T cells, allowing malignancies to evade immune surveillance (Liu et al., 2023; Li et al., 2025). While LPS activates surface receptors, translocated bacterial DNA interacts with intracellular sensors such as Toll-Like Receptor 9 (TLR9) and the cGAS/STING pathway, leading to liver inflammation and fibrosis (Liu and Yang, 2023; Ohtani et al., 2023; Wang et al., 2025c). Unlike inert DNA, living translocated bacteria such as *K. pneumoniae* actively utilize virulence factors (e.g., PBP1B) to connect with host receptors and avoid elimination, resulting in the creation of a pro-tumorigenic niche (Liu et al., 2023; Wang et al., 2025a).

### 2.9 PAMPs and inflammation (LPS/TLR4 signaling)

**Figure 1:**
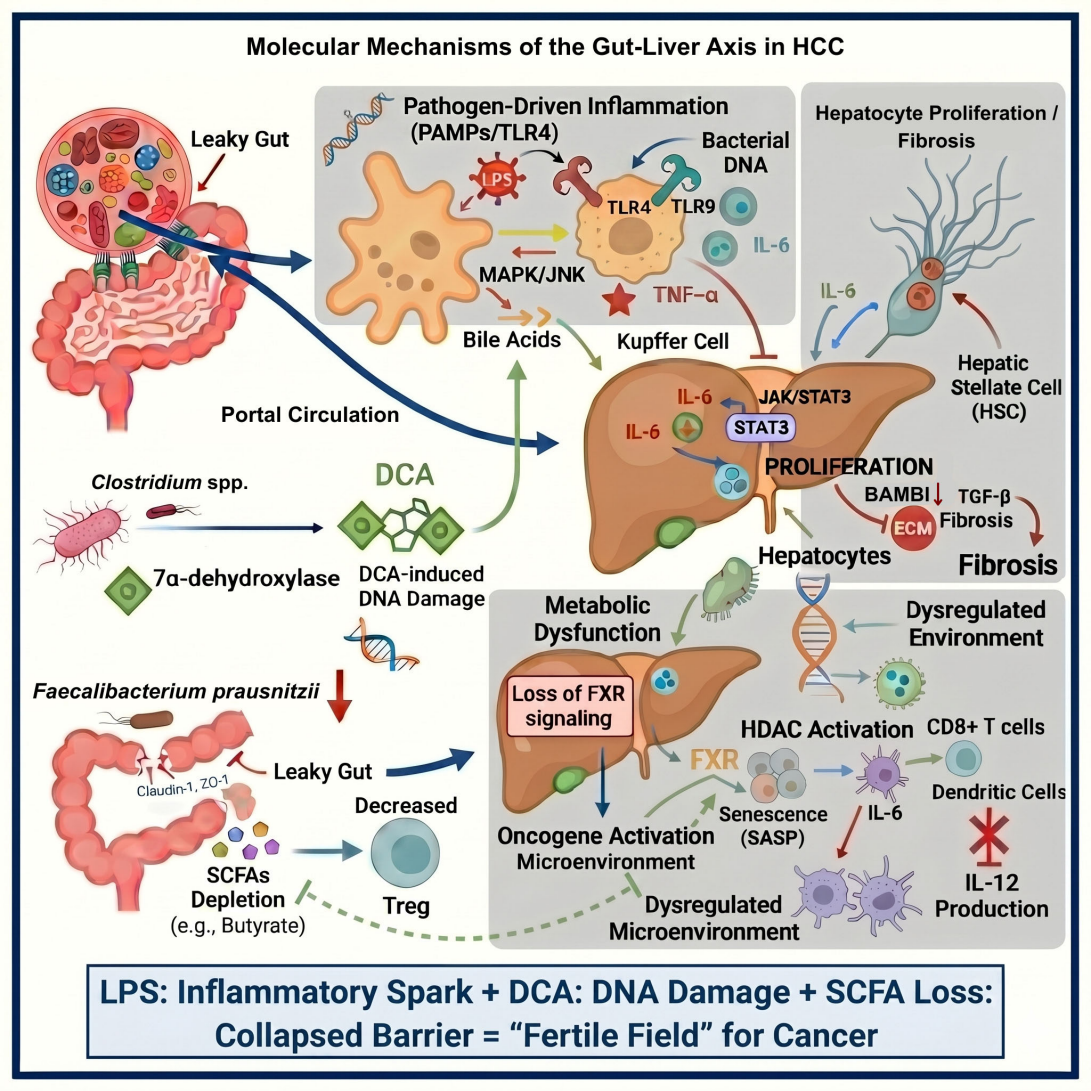
Molecular mechanisms of the gut-liver axis in HCC. Gut dysbiosis and the depletion of protective short-chain fatty acids (SCFAs, e.g., butyrate) lead to decreased regulatory T cells (Tregs) and initiate a “leaky gut.” This compromised barrier allows pathogen-associated molecular patterns (PAMPs), including bacterial LPS and DNA, to enter the portal circulation. In the liver, LPS activates TLR4 on Kupffer cells (triggering MAPK/JNK signaling and TNF-α release), while bacterial DNA activates TLR9 (releasing IL-6). Concurrently, Clostridium spp. convert primary bile acids into toxic deoxycholic acid (DCA), which induces DNA damage. These inflammatory signals drive hepatocyte proliferation via the JAK/STAT3 pathway, while Hepatic Stellate Cells (HSCs), TGF-β, and downregulated BAMBI promote extracellular matrix (ECM) deposition and fibrosis. The resulting dysregulated microenvironment is characterized by the loss of FXR signaling, cellular senescence (SASP) releasing further IL-6, HDAC activation suppressing CD8+ T cells, and impaired IL-12 production by dendritic cells. Together, this inflammatory spark, DNA damage, and collapsed barrier create a “fertile field” for hepatocarcinogenesis.

The molecular cause of HCC frequently begins with gut dysbiosis, which weakens the intestinal mucosal barrier in a process described as “leaky gut” (Fu et al., 2025). This disruption permits LPS from Gram-negative bacteria to enter the portal circulation (Xiao et al., 2025), making the liver the first organ exposed to these toxic factors (Shen et al., 2022) (**Figure 1**. Shows the molecular pathogenesis of the gut-liver axis in HCC). The molecular pathogenesis involves several key steps; LPS is a powerful inflammatory trigger that interacts with Pattern Recognition Receptors (Wang et al., 2025c), primarily binding to TLR4 expressed on resident Kupffer cells (Liu and Yang, 2023; Chen Z. et al., 2025) and hepatic stellate cells (HSCs) (Li and Liu, 2025). TLR4 binding activates intracellular signaling cascades, most notably degrading inhibitory proteins to allow the nuclear factor-κ B (NF-κB) pathway (Xiao et al., 2025) and MAPK/JNK signaling pathways (Luo et al., 2022a) to enter the nucleus. These pathways cause Kupffer cells to release pro-inflammatory cytokines, including TNF-α and IL-6 (Marawan et al., 2025). IL-6 acts as a key mediator triggering the JAK/STAT3 signaling pathway in hepatocytes (Song Y. et al., 2023), leading to chronic hepatic inflammation, oxidative damage, and the release of reactive oxygen species (ROS) (Luo et al., 2022a; Wang et al., 2025c). The production of these cytokines creates a “fertile field” for cancer (Xiao et al., 2025). The IL-6/STAT3 axis prevents apoptosis and promotes compensatory proliferation of damaged hepatocytes (Fu et al., 2025), while sustained inflammatory signals recruit immunosuppressive cells, such as MDSCs (Luo et al., 2022a). TLR4 activation in HSCs triggers the overexpression of epiregulin, a strong hepatomitogen that suppresses programmed cell death in hepatocytes (Marawan et al., 2025; Ren et al., 2025). Furthermore, LPS-TLR4 signaling enhances HSC transition into myofibroblasts and boosts TGF-β signaling by blocking the negative regulator BAMBI, directly promoting fibrosis and carcinogenesis (Marawan et al., 2025).

### 2.10 Metabolites: bile acids and SCFAs

The enzyme 7-α-dehydroxylase is expressed by some gut bacteria, including *Clostridium* clusters XI and XIVa (Ye et al., 2025). This enzyme converts primary BAs to secondary BAs, such as DCA (Daniel et al., 2024). Dysbiosis causes an excessive synthesis of DCA, which is then reabsorbed into the liver (Ohtani et al., 2023). High amounts of DCA serve as a carcinogen by producing ROS and causing oxidative stress (Zhang et al., 2024). DCA inhibits mitochondrial activity and stimulates enzymes such as COX, which raises ROS levels (Luo et al., 2022a). The buildup of ROS produces direct DNA damage and genomic instability in liver cells (Li and Liu, 2025). Preclinical models indicate that the DNA damage generated by DCA induces senescence in HSCs (Li et al., 2025). DCA works with lipoteichoic acid (LTA) to trigger TLR2 signaling in HSCs (Xu et al., 2021). This cooperative route activates Cyclooxygenase-2 (COX-2), a rate-limiting enzyme in prostaglandin synthesis (Feng et al., 2025). Senescent HSCs develop the SASP and produce pro-inflammatory cytokines and chemokines (Rajapakse et al., 2023). This pathway’s major output is prostaglandin E2 (PGE2), which inhibits the antitumor immune response (Ohtani et al., 2023). PGE2 suppresses the activity of CD8+ T lymphocytes and dendritic cells, resulting in an immunosuppressive microenvironment (Feng et al., 2025). In contrast, the Farnesoid X Receptor (FXR) functions as a “tumor suppressor” by controlling bile acid balance and production (Zhang et al., 2024). FXR inhibits several carcinogenic pathways, including the Wnt/β-catenin and JAK/STAT3 cascades (Wang et al., 2025b). It also suppresses the transcription of oncogenes including *MYC* and Cyclin D1 (Luo et al., 2022a). Dysbiosis, on the other hand, reduces FXR expression via inflammation-mediated suppression (Rajapakse et al., 2023). Loss of FXR causes a buildup of cytotoxic BAs, which induces *c-Myc* expression (Luo et al., 2022b). Furthermore, FXR deficiency stimulates the oncogenic driver YAP, resulting in prolonged activation of the Wnt pathway (Wu et al., 2021; Wang et al., 2025b).

*Faecalibacterium prausnitzii* and other butyrate-producing species are much lower in HCC patients (Xu et al., 2021). This dysbiosis significantly reduces the physiological quantities of butyrate accessible to the host (Jinato et al., 2024). Butyrate is a strong histone deacetylase (HDAC) inhibitor when used normally (Zhang M. et al., 2025). When butyrate levels fall, increased HDAC activity causes epigenetic remodeling, which represses genes required for apoptosis (Wang et al., 2025c). Specifically, a shortage of butyrate permits SIRT-1 to remain elevated, inactivating the tumor suppressor p53 (Liu and Yang, 2023). Cyclin D1 and other pro-proliferative markers are upregulated when p53 is not activated (Liu and Yang, 2023). SCFAs also help to maintain the intestinal barrier by upregulating tight junction proteins such claudin-1 and ZO-1 (Ye et al., 2025). They are the primary energy source for colonocytes, lowering epithelial apoptosis (Xiao et al., 2025). Butyrate additionally stimulates goblet cells to produce mucus layers (Chen Z. et al., 2025). Furthermore, SCFAs induce the development of regulatory T cells (Tregs), which helps to maintain immunological tolerance (Li et al., 2022). SCFA depletion is the key “domino” that causes the leaky gut phenomena and bacterial translocation (Xiao et al., 2025). This gap permits PAMPs to enter the portal circulation and activate TLR4 in the liver, causing hepatocarcinogenesis (Li and Liu, 2025).

## 3 Correlation of the “dysbiosis index” with HCC progression

Dysbiosis often worsens as liver disease advances, with primary liver cancer patients having a much higher “degree of dysbiosis” (Ddys) than healthy controls (Luo et al., 2022a; Guo et al., 2026). While diversity diminishes dramatically between health and cirrhosis, a “V-shaped” pattern occurs during the transition from cirrhosis to early-stage HCC, when microbial diversity momentarily rises due to opportunistic bacterial overgrowth (Wu et al., 2021; Xu et al., 2021; Li and Liu, 2025). HCC patients with metabolic dysfunction-associated fatty liver disease (MAFLD) had around 40% less variety than simple steatosis (Guo et al., 2026). As the condition progresses from basic fatty liver to HCC, helpful genera such as *Bifidobacterium* decline, while pathogens such as *Streptococcus* and *Escherichia-Shigella* increase (Yang et al., 2023; Lee et al., 2025). Bacterial DNA in the blood and liver gradually rises throughout these stages (Monti et al., 2025). Intratumoral microbiomes also show stage-dependent dynamics, with *Sphingomonas* abundance positively correlated with advanced clinical stages (Jo et al., 2024).

## 4 Microbiome as a biomarker in HCC

### 4.1 Microbial signatures and non-invasive biomarkers

Based on research from the previous decade, our review seeks to identify distinct microbiological signatures that consistently link with HCC development (Li and Liu, 2025; Monti et al., 2025). HCC patients exhibit a “core” pattern of dysbiosis, which includes an enrichment of Proteobacteria specifically *Escherichia-Shigella* and *Klebsiella* and *Enterococcus*, as well as a depletion of beneficial commensals such as *Akkermansia muciniphila*, *Bifidobacterium*, and *Lactobacillus* (Acharya and Thirunavukkarasu, 2024; Li and Liu, 2025; Monti et al., 2025). In a discovery cohort, a diagnostic algorithm based on 30 microbial markers obtained an area under the curve (AUC) of 80.64% in detecting early HCC but these findings require large-scale external validation before clinical implementation (Xu et al., 2021; Li and Liu, 2025). This review investigates how insights into gut-liver interaction may offer potential pathways for the development of non-invasive biomarkers, such as TMAO, secondary BAs, and SCFAs (Xu et al., 2021; Rajapakse et al., 2023; Yu et al., 2024a). Finally, we explore how multi-omics models that combine microbiological data with serological barrier indicators (such as antibodies to LPS and flagellin) may eventually help predict immunotherapy response in high-risk patients, though standardized clinical assays are currently lacking (Li et al., 2022; Acharya and Thirunavukkarasu, 2024; Daniel et al., 2024).

### 4.2 Microbial biomarkers for early diagnosis and prognostication

Recent investigations have identified specific fecal bacterial signatures that show promise in early-phase studies to complement traditional markers like alpha-fetoprotein (AFP), aiming to enhance diagnostic sensitivity. In a study utilizing 16S rRNA sequencing, the phylum Actinobacteria was found to be increased in early HCC compared to cirrhosis (Kaźmierczak-Siedlecka et al., 2021). At the genus level, *Gemmiger* and *Parabacteroides* were enriched in HCC patients, whereas butyrate-producing genera were decreased and LPS-producing genera were increased relative to healthy controls (Kaźmierczak-Siedlecka et al., 2021). A diagnostic model incorporating 30 microbial markers achieved an AUC of 80.64% in distinguishing early HCC from non-HCC samples (Kaźmierczak-Siedlecka et al., 2021).

Other studies have highlighted the potential of combining oral and gut microbiota markers. One study suggested that merging 19 optimal microbial biomarkers could establish a distinct classification that increased diagnostic accuracy to 0.9405 within that specific cohort (Yang et al., 2023). Importantly, when paired with AFP levels, this combined prediction model’s accuracy further improved to 0.9811, suggesting a strong complementary effect to serum markers (Yang et al., 2023).

Specific bacterial alterations also track with disease progression. In HBV-related cases, genera such as *Streptococcus*, *Prevotella-9*, *Faecalibacterium*, and *Bacteroides* were identified as crucial elements in the progression to HCC (Yang et al., 2023). Validating this diagnostic potential, another study showed that a Probability of Disease (POD) index based on microbial markers achieved an AUC of 76.80% in distinguishing early HCC from controls (Ren et al., 2019; Yu et al., 2024b). Conversely, beneficial taxa like *Akkermansia* are reduced in HCC, while *Bacteroides* and *Ruminococcaceae* are increased (Zhang et al., 2020). *Akkermansia* and *Bifidobacterium* also show an inverse correlation with fecal calprotectin, a marker of intestinal inflammation (Zhang et al., 2020).

These microbial shifts are functionally linked to intestinal barrier integrity. HCC patients exhibit significantly elevated levels of serum LPS, ZO-1, and fecal calprotectin compared to controls, indicating enhanced intestinal permeability (Yang et al., 2023). The “Cirrhosis Dysbiosis Ratio” (CDR), which compares beneficial to pathogenic taxa, serves as a functional index; a lower CDR correlates with higher endotoxemia, Model for End-Stage Liver Disease (MELD) scores, and the likelihood of organ failure (Huo et al., 2023; Capasso et al., 2025).

## 5 Microbiome role in clinical management of HCC

### 5.1 Modulation of the gut microbiome to enhance immunotherapy efficacy

**Figure 2:**
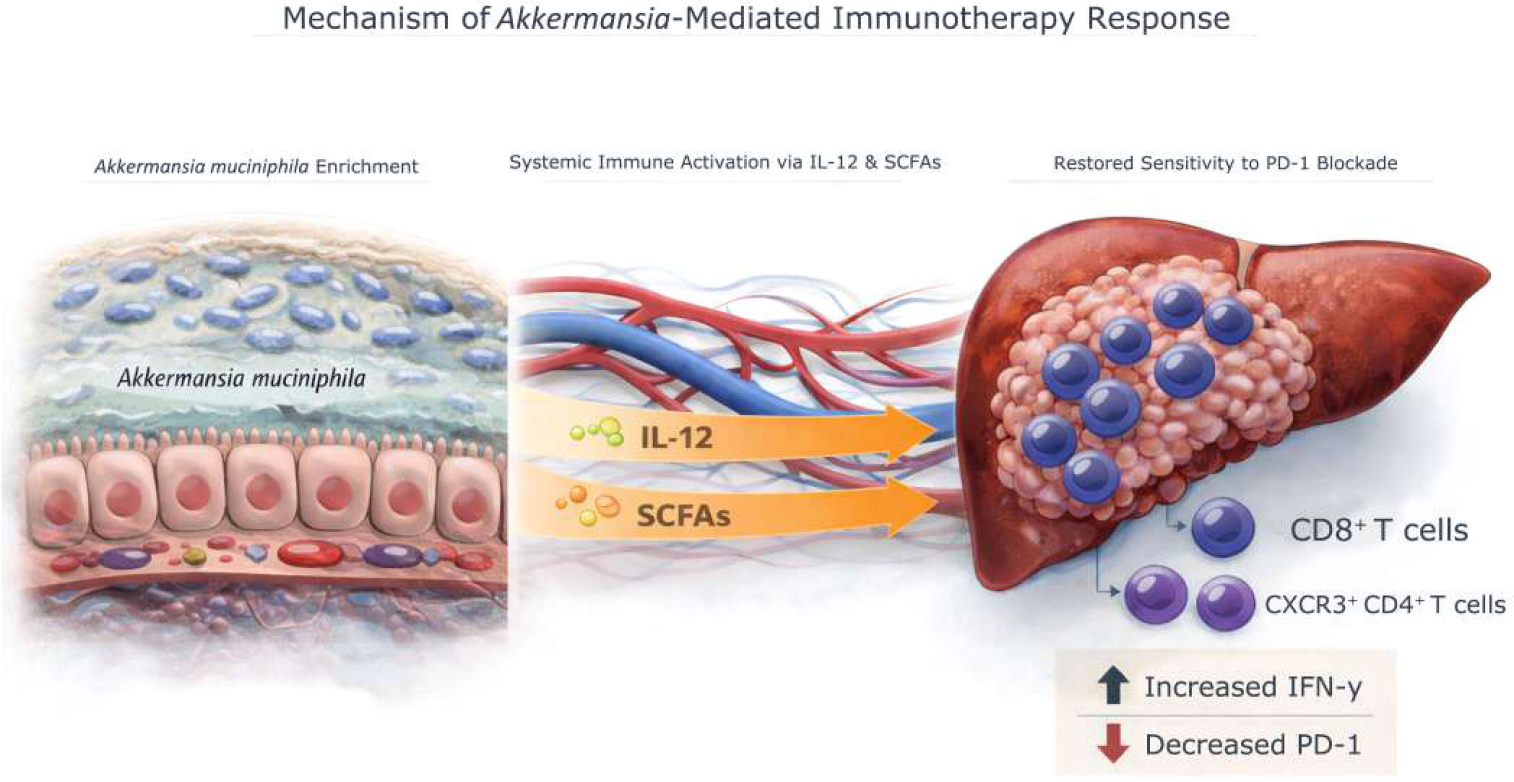
Mechanistic pathway of *Akkermansia muciniphila* in enhancing immunotherapy efficacy in HCC. 1- *Akkermansia muciniphila* colonizes the intestinal mucus layer, reinforcing barrier integrity. 2- This colonization stimulates the release of systemic mediators, including Interleukin-12 (IL-12) and Short-Chain Fatty Acids (SCFAs). 3- These signals recruit CXCR3+ CD4+ and cytotoxic CD8+ T cells into the HCC tumor microenvironment. The infiltrating T cells display an activated phenotype with increased IFN-γ production and downregulated PD-1 expression, thereby overcoming resistance to PD-1 blockade therapy.

The gut microbiome is a critical determinant of the response to ICIs. In human clinical cohorts, patients who respond to ICIs exhibit an enrichment of specific species, notably *Akkermansia muciniphila* (Chen PJ. et al., 2025). Preclinical murine models suggest that mechanistically, *Akkermansia muciniphila* promotes the recruitment of CXCR3+CD4+ and CD8+ T lymphocytes into the tumor microenvironment via Interleukin-12 (IL-12) secretion, thereby enhancing antitumor immunity (Chen PJ. et al., 2025). FMT from responders into germ-free mice has been shown to restore sensitivity to Programmed Cell Death Protein 1 (PD-1) blockade, while FMT from non-responders did not (Chen PJ. et al., 2025). In addition to *Akkermansia*, *Bifidobacterium* species reinforce the intestinal barrier and may enhance ICI effects through cross-feeding interactions that produce SCFAs (Ponziani et al., 2022) (**Figure 2**. Explains the pathway of *Akkermansia muciniphila* in enhancing immunotherapy efficacy in HCC). In preclinical models, the transplantation of a consortium of *Bacteroides acidifaciens*, *Odoribacter laneus*, and *Odoribacter splanchnicus* (BOO) inhibited HCC growth by activating the AMPK pathway and inhibiting mTOR signaling in NK and T cells (Davar et al., 2021; Pan et al., 2023; Wu et al., 2025). This modulation resulted in upregulated Interferon-gamma (IFN-γ) and downregulated PD-1 expression, rescuing immune competence (Davar et al., 2021; Pan et al., 2023; Wu et al., 2025). Additionally, FMT from healthy donors has been shown to enhance CD8+ T cell infiltration into the tumor, helping to reactivate antitumor activity (Xiao et al., 2025).

Conversely, clinical data indicates that antibiotic usage can detrimentally affect immunotherapy outcomes. A cohort study revealed that antibiotic use within 30 days of initiating ICIs was associated with a hazard ratio (aHR) of 1.65 to 1.94 for cancer-related mortality, likely due to the depletion of these beneficial immunomodulatory bacteria (Cheung et al., 2021; Qin et al., 2023).

Despite these promising results, current microbiome-immunotherapy research in HCC is limited by conflicting findings and a lack of standardized clinical validation. While *Akkermansia muciniphila* is frequently cited as a positive predictor of ICI response, other studies have reported varying results based on geographic and dietary differences (Capasso et al., 2025; Wu et al., 2025). Furthermore, recent evidence suggests that high baseline microbiome diversity does not universally guarantee therapeutic efficacy (Capasso et al., 2025; Wu et al., 2025). Immunotherapy responsiveness is likely governed by complex, community-level microbial interactions rather than the presence of single specific taxa (Capasso et al., 2025; Wu et al., 2025). Consequently, more prospective randomized controlled trials are urgently needed to critically evaluate these conflicting parameters before microbiome-based stratification can be reliably implemented in HCC immunotherapy protocols.

### 5.2 Gut-targeted therapies for HCC prevention and prophylaxis

Preclinical therapeutic strategies targeting the gut-liver axis show promise in preventing hepatocarcinogenesis in animal models. The non-absorbable antibiotic Rifaximin demonstrated protective effects in a MASH rat model by reinforcing the intestinal barrier and reducing portal LPS translocation (Nishimura et al., 2025). Rifaximin treatment suppressed the LPS-TLR4 cascade, thereby attenuating the expression of the hepatomitogen epiregulin and pro-inflammatory cytokines like IL-8 (Nishimura et al., 2025).

Next-Generation Probiotics (NGPs) such as *Faecalibacterium prausnitzii* and *Clostridium butyricum* are also being explored in early-phase and preclinical settings. In murine models of liver injury, strains of *F. prausnitzii* (e.g., LB8, ZF21) were shown to upregulate hepatic antioxidative enzymes (GSH-PX, SOD) and reduce lipid peroxidation markers like MDA (Zhu et al., 2024). These effects are largely mediated by butyrate, which enhances barrier function and activates the Nrf2 antioxidative pathway (Zhu et al., 2024).

### 5.3 Management of treatment-related toxicity

Emerging evidence from preliminary studies suggests that the gut microbiota may play a role in the efficacy and toxicity of Tyrosine Kinase Inhibitors (TKIs) (**Table 2**. Establish the therapeutic strategies targeting the gut microenvironment). In the context of Epidermal Growth Factor Receptor (EGFR)-TKIs, patients with a “partial response” had a significantly higher abundance of *Ruminococcus* compared to those with stable or progressive disease (Tabe et al., 2026). A *Ruminococcus* abundance cutoff of 1.4% was predictive of treatment efficacy (Tabe et al., 2026). Additionally, in animal models of afatinib-induced diarrhea, administration of sitagliptin altered the gut microbiota composition (increasing *Akkermansia* and *Coriobacteriaceae*) via GLP-2 pathways, suggesting a potential avenue for managing TKI-associated gastrointestinal toxicity (Ariyadamrongkwan and Muanprasat, 2025).

**Table 2:** Therapeutic strategies targeting the gut microenvironment.

Therapeutic category	Agent/strategy	Mechanism of action	Application in gut-liver management	Evidence tier/model	References
Probiotics/Postbiotics	*Lactobacillus rhamnosus* GG (LGG), p40 protein	The secreted protein p40 stimulates intestinal epithelial cells to release HB-EGF, which activates EGFR-Akt signaling. This prevents cytokine-induced apoptosis, promotes cell survival, and restores tight junction distribution.	Strengthening the intestinal barrier to reduce the translocation of endotoxins (LPS) to the liver.	Preclinical (*In vivo* murine models & *In vitro*)	(Ariyadamrongkwan and Muanprasat, 2025)
Intestinal Hormones	GLP-2 (Glucagon-like peptide-2)	Promotes crypt cell proliferation, increases villus height, and aids epithelial repair through ERBB-dependent signaling. It alleviates villus atrophy and crypt apoptosis caused by targeted therapies.	Enhancing gut repair mechanisms to maintain mucosal integrity during systemic cancer treatment.	Preclinical (*In vivo* murine models)	(Ariyadamrongkwan and Muanprasat, 2025)
Channel Inhibitors	Crofelemer, CaCCinh-A01	Inhibits chloride channels, specifically calcium-activated chloride channels (CaCCs) and CFTR, to abolish excessive fluid secretion and reduce stool water content.	Managing secretory symptoms and fluid loss in advanced cancer to prevent dehydration and electrolyte imbalance.	Human Clinical Trials & Preclinical	(Ariyadamrongkwan and Muanprasat, 2025)
Herbal Medicine	Hangeshashinto (HST)	Inhibits CaCCs to reduce fluid secretion and contain bioactive compounds (e.g., baicalein, ginsenoside, 6-shogaol) that exert anti-inflammatory effects.	A multi-target approach to simultaneously reduce gut inflammation and manage secretory toxicity.	Human Clinical Trials & Preclinical	(Ariyadamrongkwan and Muanprasat, 2025)
Anti-diabetic Agents	Sitagliptin (DPP-4 Inhibitor)	Increases GLP-2 levels by inhibiting its degradation, which improves the expression of tight junction proteins (ZO-1, occludin, E-cadherin) and increases anti-inflammatory cytokines.	Repurposing existing drugs to support gut barrier function and reduce gut-derived inflammation affecting the liver.	Preclinical (*In vivo* murine models)	(Ariyadamrongkwan and Muanprasat, 2025)
Microbiota Biomarkers	*Ruminococcus* abundance	Higher abundance of *Ruminococcus* correlates with better partial response (PR) to treatment, and higher α-diversity is associated with less severe diarrhea.	Monitoring specific gut taxa (*Ruminococcus*) as a biomarker for treatment efficacy and gut homeostasis.	Human Clinical Cohorts (Observational)	(Tabe et al., 2026)

### 5.4 Perioperative and post-surgical management

The gut microbiome influences recovery following liver resection. In human clinical cohorts, a higher abundance of *Bifidobacterium longum* is significantly correlated with timely liver function recovery and shorter postoperative hospital stays (Yu et al., 2024b). In contrast, intratumoral microbiome analysis identifies high-risk profiles; for instance, a greater abundance of *Intestinimonas* predicts a worse prognosis (HR = 2.082), whereas *Brachybacterium* and *Rothia* are associated with better outcomes (Jiang et al., 2024).

Regarding liver transplantation, gut microbiota modulation may attenuate ischemia-reperfusion injury (IRI). Fecal transplantation from antibiotic-pretreated mice into germ-free recipients resulted in reduced liver damage and necrosis following IRI compared to controls, indicated by lower serum ALT and cleaved caspase-3 levels (Lu et al., 2023; Wang et al., 2024). For immunocompromised patients undergoing FMT, strict safety protocols are essential. According to current protocols, donor stool must be screened for infectious agents and processed under good manufacturing practice (GMP) conditions to prevent the transmission of multi-drug-resistant organisms (Pomej et al., 2025).

While FMT and next-generation probiotics hold great potential for therapeutic translation, this is subject to strict regulatory and ethical challenges. The FDA and EMA consider FMT as an investigational biological drug that must go through strict and lengthy approval procedures (Ponziani et al., 2022; Chen PJ. et al., 2025; Pomej et al., 2025). In addition, standardization of these living therapies comes with significant logistical and ethical issues, such as the extensive screening needed to find a ‘healthy’ donor (Ponziani et al., 2022; Chen PJ. et al., 2025; Pomej et al., 2025). The dynamic nature of the microbiome also poses a risk of unintentionally transferring undetected pathogens, antimicrobial-resistant genes or pro-oncogenic metabolites to immunosuppressed HCC patients (Ponziani et al., 2022; Chen PJ. et al., 2025; Pomej et al., 2025). As a result, therapeutic strategies are moving towards synthetic defined microbes and sterile postbiotics to avoid donor-related risks and adhere to strict safety standards.

## 6 Future perspectives

While the diagnostic and therapeutic potential of the microbiome-onco axis in HCC is undeniable, its immediate integration into routine clinical practice is currently hindered by several methodological bottlenecks (Attia et al., 2024). Most existing investigations rely on relatively small, geographically restricted cohorts with retrospective designs, which limits the generalizability and reproducibility of identified microbial signatures. Furthermore, the significant heterogeneity among currently available reports driven by inherent confounding factors such as varied dietary patterns, polypharmacy, and concurrent metabolic comorbidities often obscures true microbiome-tumor associations and complicates the synthesis of consistent cross-study conclusions. This is compounded by the fact that a patient’s baseline microbiome composition is deeply dictated by geographic location, cultural dietary habits, obesity, alcohol intake, and historical antibiotic exposure. This inter-study variability is further exacerbated by differences in disease staging and the inclusion of diverse patient etiologies, ranging from viral hepatitis to MASLD, which prevents the universal application of specific microbial signatures. To overcome these limitations, future initiatives must prioritize large-scale, multi-center, and prospective longitudinal cohorts to robustly validate non-invasive microbial biomarkers across diverse global populations. Beyond patient-related variables, a primary obstacle to clinical integration is the lack of standardized methodologies for microbiome analysis. Currently, there is no optimal sample collection technique, with significant biological differences observed between fecal samples and mucosal-associated microbiota (Attia et al., 2024; Wang et al., 2026). Furthermore, variations in DNA extraction kits and storage conditions can inadvertently skew microbial profiles (Attia et al., 2024; Zoruk et al., 2025). The choice of sequencing platform also introduces bias; while 16S rRNA sequencing is cost-effective for taxonomic profiling, it lacks the resolution of shotgun metagenomics, which provides essential functional insights into microbial metabolic pathways (Attia et al., 2024; Zoruk et al., 2025; Kapoor et al., 2026). Finally, the use of disparate bioinformatic pipelines and reference databases for data processing further complicates the comparability of results across different research centers (Kapoor et al., 2026; Wang et al., 2026). Establishing international protocols across these technical domains is essential to ensure the reproducibility and diagnostic reliability required for precision oncology (Attia et al., 2024; Zoruk et al., 2025; Kapoor et al., 2026; Wang et al., 2026). Beyond these scientific and technical challenges, pragmatic logistical challenges such as high financial costs of high-throughput sequencing, long bioinformatic turnaround times and a critical shortage of CLIA-certified laboratories able to perform these complex assays, limit the routine clinical use of microbiome biomarkers.

Moving forward, the field must transition from descriptive, taxonomy-based analyses (such as 16S rRNA sequencing) toward comprehensive, functional multi-omics frameworks. Future advancements will heavily rely on integrating high-resolution shotgun metagenomics with metatranscriptomics, metabolomics (profiling circulating SCFAs and secondary bile acids), and host genomic or epigenomic data (Dakal et al., 2025). Decoding this unprecedented volume of high-dimensional data will necessitate the advanced deployment of artificial intelligence (AI) and machine learning (ML) algorithms. By training AI models to synthesize these complex biological networks alongside standard clinical features, researchers can uncover highly accurate, dynamic algorithms for early HCC prediction, disease prognostication, and the stratification of patients for targeted immunotherapies (Ghosh et al., 2025).

Ultimately, a critical limitation of current human microbiome research in HCC is its predominantly observational nature, which establishes association rather than direct causation. To bridge this gap between correlation and causation, the utilization of advanced pre-clinical models, such as germ-free mice colonized with patient-derived consortia and cutting-edge liver-on-a-chip technologies, will be vital for definitively proving the mechanistic roles of specific bacterial strains and their metabolic outputs (Guo et al., 2023; Hu et al., 2025). In the therapeutic arena, we anticipate a paradigm shift toward “precision microbiome engineering”. This will likely include the development of next-generation, genetically engineered probiotics designed to deliver localized immunomodulatory or barrier-enhancing payloads directly to the gut mucosa (Muhammad et al., 2026). By transitioning from generalized interventions to tailored FMT and customized postbiotic therapies based on a patient’s baseline enterotype, the modulation of the gut-liver axis can be firmly embedded into the precision oncology toolkit for HCC management.

## 7 Conclusion

The evolving understanding of the gut-liver axis has firmly established the microbiome as a central driver of HCC pathogenesis, particularly within the rapidly expanding demographic of MASLD. As detailed in this review, dietary triggers and functional dysbiosis consistently precipitate a “leaky gut”, facilitating the unchecked translocation of pathogen-associated molecular patterns and toxic metabolites that ignite chronic, oncogenic metainflammation and immune evasion. Translating these mechanistic insights into the clinic offers profound diagnostic and therapeutic opportunities; distinct microbial signatures present a highly promising avenue for non-invasive early detection, circumventing the limitations of traditional ultrasound, while the targeted modulation of the gut microbiome could critically enhance the efficacy of ICIs. Although integrating these findings into routine practice necessitates advanced, multi-omics validation to overcome existing methodological bottlenecks, restoring host-microbiome homeostasis represents a transformative, precision-oncology frontier for combating the escalating global burden of HCC.
